# Effect of gelatin-polysuccinat on cerebral oxygenation and microcirculation in a porcine haemorrhagic shock model

**DOI:** 10.1186/s13049-018-0477-2

**Published:** 2018-02-09

**Authors:** Alexander Ziebart, Christian Möllmann, Andreas Garcia-Bardon, Jens Kamuf, Moritz Schäfer, Rainer Thomas, Erik K. Hartmann

**Affiliations:** Department of Anaesthesiology, Medical Centre of the Johannes Gutenberg-University, Langenbeckstraße 1, 55131 Mainz, Germany

**Keywords:** gelatin-polysuccinat, haemorrhagic shock, fluid resuscitation, cerebral oxygen saturation, pig model

## Abstract

**Background:**

During early treatment of haemorrhagic shock maintenance of cerebral and end-organ oxygen supply by fluid resuscitation is mandatory. Gelatin-polysuccinat (GP) recently regained attention despite a still unclear risk profile and widely unknown effects on cerebral and peripheral microcirculation. This study investigates the effects of GP versus balanced electrolyte solution (BEL) with focus on cerebral regional oxygen saturation and peripheral microcirculation in a porcine haemorrhagic shock model.

**Methods:**

After Animal Care Committee approval haemorrhagic shock was induced by arterial blood withdrawal in 27 anaesthetized pigs. Consequently, the animals received rapid fluid resuscitation by either GP or BEL to replace the removed amount of blood, or remained untreated (*n* = 3 × 9). Over two hours cerebral regional oxygen saturation by near-infrared spectroscopy and peripheral buccal microcirculation by combined white-light spectrometry and laser-Doppler flowmetry were recorded. Secondary parameters included extended haemodynamics, spirometry, haematological and blood gas parameters.

**Results:**

Both fluid resuscitation regimes sufficiently stabilized the macro- and microcirculation in haemorrhagic shock with a more pronounced effect following GP infusion. GP administration led to a persisting, critical impairment of cerebral regional oxygen saturation through considerable haemodilution. Survival rates were 100% in both fluid resuscitation groups, but only 33% in the untreated control.

**Conclusion:**

Equal amounts of GP and BEL sufficiently stabilize systemic circulation and microcirculatory perfusion. Forced fluid resuscitation by GP should be applied with caution to prevent haemodilution-induced impairment of cerebral oxygen delivery.

## Background

Fast and efficient therapy of haemorrhagic shock is one of the most challenging tasks in prehospital and early clinical emergency medicine. Massive blood loss can be observed in more than 15% of all injuries resulting in death [1, 2]. The main reason is circulatory collapse, which leads to insufficient end organ oxygen supply and can induce irreversible organ failure [3]. The brain in particular can only compensate hypoxaemia for short periods [4]. Moreover, the resulting inflammation response has the potential to cause further injury leading to multiple organ failure [5, 6]. In prehospital or early clinical treatment of severely injured patients, before availability of blood transfusion or surgical bleeding control, different fluid resuscitation regimes based on crystalloids and colloids are applied in order to prevent cardio-circulatory failure [6, 7]. Colloids, in this context, exert higher volume effects due to the macromolecule-related increase of the oncotic pressure, whereas crystalloids tend to shift into the extravascular space. Hence, colloids are thought to induce a longer lasting and more effective volume effect leading to macrocirculatory stabilisation [8]. For many years, colloids and especially hydroxyethyl starch (HES) were regarded as standard treatment in various shock conditions induced by relative or absolute hypovolaemia. Recently several studies questioned the safety and benefit of HES in critically ill and especially septic patients [9–11]. Negative side effects include renal failure with higher rates of renal replacement therapy and mortality [12–14]. Other studies contradict these results, describe benefits for the patients and refuse the increase in mortality rates [14, 15]. Nevertheless, this led to a negative risk assessment by the European Medicines Agency, and resulted in permission to use HES being temporarily revoked. Consequently, the use of HES products was prohibited in patients suffering from systemic sepsis or severe burn injury. Recent guidelines on perioperative fluid management re-approved the use of different colloidal solutions for acute hypovolaemia without detecting clear evidence for any particular substance outside the upper mentioned limitations [6]. Gelatin-polysuccinat (GP) is a relatively old and well-known colloid that consists of gelatin polypeptides derived from bovine collagen. For years GP was only regarded as a secondary option for treatment of hypovolaemic shock behind modern HES solutions, which might be explained by the highest rates of anaphylactic reactions among all colloids through GP with an incidence of 0.05–0.1% [16, 17]. While the haemodynamic effects are comparable between both solutions, only the intravascular persistence of GP seems to be shorter [18, 19]. Despite regaining focus for treatment of acute hypovolaemia during the aforementioned controversy, the number of studies, which investigate the disadvantages and advantages of GP in distinct clinical scenarios are limited [20–23]. Urgent re-establishment of adequate microcirculatory, end organ and especially cerebral oxygen supply represent the cornerstones in acute hypovolaemia or haemorrhagic shock. Therefore, we hypothesized that GP stabilizes the cerebral oxygenation measured by near-infrared spectroscopy-derived cerebral regional oxygen saturation (crSO_2_) more sufficiently than common balanced electrolyte solution (BEL) in a porcine model of haemorrhagic shock by high-volume blood withdrawal. Secondary outcome parameters include assessment of the peripheral microcirculation and extended cardio-circulatory monitoring.

## Methods

### Anaesthesia and instrumentation

The study was conducted after approval of the State and Institutional Animal Care Committee (Landesuntersuchungsamt Rheinland-Pfalz, Koblenz, Germany; Chairperson: Dr. Silvia Eisch-Wolf; reference number: 23,177–07/G 14–1-084; 02.02.2015) from 02/2015–11/2015 and in accordance with the ARRIVE guidelines. 27 juvenile, male pigs (*Sus scrofa domestica*; mean weight 29 ± 1 kg; age: two months) were included in a prospective study: each nine animals received fluid resuscitation by means of either GP or BEL, respectively. Nine animals remained untreated (control group; no fluid resuscitation).

To minimize stress, the animals stayed in their known environment for as long as possible. A local breeder maintained their overall condition. After intramuscular injection of ketamine (8 mg kg^− 1^) and midazolam (0.2 mg kg^− 1^) the sedated animals were delivered to the laboratory. After being placed in the supine position, fentanyl (4 μg kg^− 1^) and propofol (4 mg kg^− 1^) were injected intravenously to induce general anaesthesia. Throughout the entire experiment, anaesthesia was maintained by continuous infusion of fentanyl (0.1–0.2 mg h^− 1^) and propofol (8–12 mg kg^− 1^ h^− 1^). A single dose of atracurium (0.5 mg kg^− 1^) was administered to facilitate endotracheal intubation. The animals were ventilated in volume-controlled mode (AVEA, CareFusion, USA): tidal volume 8 ml kg^− 1^, positive end-expiratory pressure of 5 cmH_2_O, fraction of inspired oxygen of 0.4, inspiration to expiration ratio 1:2, and variable respiration rate to achieve an end-tidal CO_2_ < 6 kPa. With ultrasound guidance, four femoral vascular catheters were placed as follows: central venous line (drug administration, left femoral vein); pulse contour cardiac output system (PiCCO, Pulsion Medical Systems, Germany, right femoral artery); arterial line (blood withdrawal for shock model, left femoral artery); and large-bore venous introducer (fluid resuscitation, right femoral vein). Thereby we ensured to place the PiCCO and the central venous line for thermodilution-related cold saline application into different sides. Haemodynamic and spirometric parameters were permanently measured and recorded (S5, GE Healthcare, USA) Single-indicator transpulmonary thermodilution technique was used to measure cardiac output, global enddiastolic volume and extravascular lung water. Tables 1 and 2 summarize all investigated cardiorespiratory parameters.

**Table 1:** Extended haemodynamic and blood gas parameters

Parameter Mean (SD)		BLH	Shock	0 h	1 h	2 h
number of animals	GP	9	9	9	9	9
	BEL	9	9	9	9	9
	Control	9	3	3	3	3
[mmHg]	GP	73 (12)	34 (18)*	86 (12)*#^2^	78 (15)#^2^	74 (15)
	BEL	72 (9)	27 (3)*	84 (10)*#^3^	68 (10)	65 (8)2
	Control	66 (12)	31 (3)*	48 (6)*#^2,3^	58 (12)#^1^	61 (11)
HR [min^−1^]	GP	70 (16)	101 (37)*	108 (29)*#^2^	103 (41)*#^2^	97 (39)*#^2^
	BEL	77 (28)	143 (60)*	102 (34)*#^3^	109 (46)*#^3^	120 (51)*
	Control	80 (8)	151 (36)*	170 (38)*#^2,3^	172 (60)*#^2,3^	172 (68)*#^2^
CO [%]	GP	100	60 (20)*	178 (34)*#^1,2^	155 (19)*#^1,2^	136 (22)*#^1,2^
	BEL	100	57 (13)*	130 (22)*#^1,3^	105 (16)#^1^	105 (14)#^1^
	Control	100	59 (21)*	81 (24)#^2,3^	84 (31)#^2^	90 (25)#^2^
EVLWI [%]	GP	100	97 (18)	100 (21)	101 (17)	95 (12)
	BEL	100	103 (11)	98 (9)	97 (16)	98 (18)
	Control	100	108 (34)	90 (10)	81 (9)	84 (14)
GEDI [%]	GP	100	64 (7)*	114 (10)*#^1,2^	96 (38)*#^2^	102 (15)#^1,2^
	BEL	100	67 (11)*	84 (32)#^1^	83 (16)	84 (13)#^1^
	Control	100	62 (7)*	67 (4)#^2^	65 (14)#^2^	68 (16)#^2^
CVP [mmHg]	GP	8 (3)	4 (3)*	11 (2)*#^1,2^	8 (2)	7 (1)
	BEL	8 (2)	4 (1)*	8 (2)#^1^	6 (1)*	6 (2)*
	Control	8 (2)	5 (1)	9 (2)#^2^	5 (2)	5 (3)
SpO_2_ [%]	GP	100 (1)	97 (4)	98 (2)	99 (2)	99 (1)
	BEL	100 (0)	100 (0)	100 (0)	100 (0)	100 (0)
	Control	100 (0)	100 (0)	100 (0)	100 (0)	100 (0)
SvO_2_ [%]	GP	77 (8)	43 (21)*#^1,2^	75 (11)#^1,2^	68 (10)#^1,2^	62 (11)*#^2^
	BEL	72 (14)	31 (14)*#^1^	60 (10)*#^1,3^	52 (14)*#^1,3^	54 (9)*#^3^
	Control	72 (8)	18 (8)*#^2^	27 (8)*#^2,3^	34 (4)*#^2,3^	38 (7)*#^2,3^
pH	GP	7.46 (0,04)	7.39 (0,09)	7.39 (0,04)	7.47 (0,08)	7.45 (0,08)
	BEL	7.49 (0,05)	7.45 (0,05)	7.41 (0,06)	7.47 (0,06)	7.49 (0,05)
	Control	7.57 (0)	7.54 (0,04)	7.47 (0,06)	7.47 (0,06)	7.49 (0,06)
BE [mmol/l]	GP	6 (4)	1 (4)	2 (3)	7 (2)	5 (3)
	BEL	6 (3)	2 (3)	3 (3)	6 (3)	7 (3)
	Control	9 (0)	7 (1)	5 (3)	5 (6)	7 (5)
PaCO_2_ [mmHg]	GP	42 (5)	44 (10)	45 (8)	44 (8)	46 (10)
	BEL	38 (3)	38 (2)	44 (3)*	41 (3)	40 (3)
	Control	36 (2)	37 (4)	42 (4)	41 (2)	40 (1)
avCO_2_ diff [mmHg]	GP	6 (2)	10 (4)*	9 (3)#^2^	6 (3)#^2^	10 (10)
	BEL	6 (3)	12 (3)*	9 (2)#^3^	8 (3)#^3^	8 (3)
	Control	6 (4)	15 (3)*	18 (3)*#^2,3^	18 (3)*#^2,3^	13 (3)*
avO_2_ diff [mmHg]	GP	142 (17)	134 (57)	130 (14)	139 (14)	123 (32)
	BEL	143 (17)	142 (39)	146 (15)	154 (17)	146 (21)
	Control	143 (8)	131 (25)	130 (29)	153 (11)	155 (18)
lactate [mmol/l]	GP	1,2 (0,5)	2,8 (1,5)*	2,9 (1,1)*	1,5 (0,6)	0,9 (0,3)
	BEL	1,3 (0,5)	3,2 (1,2)*	2,8 (0,9)*	1,4 (0,4)	1,1 (0,3)
	Control	1 (0,1)	3,2 (0,7)*	3,3 (0,)*	2,1 (0,6)	1,8 (0,5)
PaO_2_ [mmHg]	GP	191 (16)	185 (28)	180 (28)	181 (11)	166 (25)*
	BEL	190 (16)	166 (40)*	187 (18)	190 (20)	182 (20)
	Control	166 (23)	151 (34)	178 (7)	177 (12)	184 (15)
PaO_2_/FiO_2_ [mmHg]	GP	472 (42)	417 (140)	455 (67)	414 (115)	411 (69)
	BEL	474 (40)	415 (99)	468 (45)	475 (50)	454 (51)
	Control	465 (7)	380 (70)	395 (67)	460 (28)	459 (38)
P_peak_ [cm H_2_O]	GP	17 (3)	17 (2)	18 (3)#^2^	19 (3)#^2^	19 (2)#^2^
	BEL	17 (2)	19 (2)	17 (1)#^3^	17 (1)#^3^	18 (2)#^3^
	Control	18 (0)	19 (1)	23 (8)*#^2,3^	24 (7)*#^2,3^	24 (7)*#^2,3^
P_mean_ [cm H_2_O]	GP	8 (1)	8 (1)	8 (1)	8 (1)	8 (1)
	BEL	9 (1)	9 (1)	9 (1)	9 (1)	9 (0)
	Control	9 (0)	10 (1)	9 (1)	9 (1)	9 (1)
PEEP [cm H_2_O]	GP	4 (1)	4 (1)	4 (1)	4 (1)	4 (1)
	BEL	4 (0)	4 (0)	4 (0)	4 (0)	4 (0)
	Control	4 (0)	4 (0)	4 (0)	4 (0)	4 (0)
AaDO_2_ [mmHg]	GP	48 (18)	70 (51)	57 (18)	72 (50)	69 (29)
	BEL	51 (14)	75 (40)	47 (17)	47 (20)	56 (20)
	Control	57 (2)	90 (24)	78 (31)	54 (14)	54 (16)
MV [l/min]	GP	5.2 (1.2)#^1,2^	5.1 (1.1)#^1,2^	5.5 (1.6)#^1^	5.7 (0.8)	5.6 (1)#^1,2^
	BEL	7.0 (1.0)#^1^	6.9 (0.8)#^1^	6.9 (0.8)#^1^	6.9 (0.8)	6.9 (0.8)#^1^
	Control	7.0 (0.2)#^2^	6.8 (0.5)#^2^	6.8 (0.5)	7.2 (0.8)	7.2 (0.8)#^2^

*MAP* mean arterial pressure, *HR* heart rate, *CO* cardiac output, *EVLWI* extravascular lung water index, *GEDI* global endiastolic volume index, *CVP* central venous pressure, *SpO*_*2*_ peripheral oxygen saturation, *SvO*_*2*_ central venous saturation, *BE* base excess, *PaCO*_*2*_ arterial partial pressure of carbon dioxide, *avCO*_*2*_*diff* arteriovenous carbon dioxide difference, *avO*_*2*_*diff* ateriovenous oxygen difference, *PaO*_*2*_ arterial partial pressure of oxygen, *PaO*_*2*_*/FiO*_*2*_ Horovitz index, *P*_*peak*_ peak inspiratory pressure, *P*_*mean*_ mean airway pressure, *PEEP* positive end-expiratory pressure, *AaDO*_*2*_ alveolar-arterial oxygen difference, *MV* respiratory minute volume

*indicates *p* < 0.05 vs. Baseline value. # indicates *p* < 0.05 in intergroup comparison (1 = colloid vs. BEL; 2 = colloid vs. sham; 3 = BEL vs. sham)

**Table 2:** Cerebral saturation and peripheral microcirculatory assessment (% of baseline)

Parameter MEAN (SD)		BLH	Shock	0 h	1 h	2 h
number of animals	GP	9	9	9	9	9
	BEL	9	9	9	9	9
	Control	9	3	3	3	3
crSO_2_ [%]	GP	100 (0)	74 (15)*	82 (12)*#^1^	89 (10)*#^1^	85 (7)*#^1,2^
	BEL	100 (0)	75 (10)	99 (13)#^1^	102 (14)	101 (13)#^1^
	Control	100 (0)	80 (11)	92 (11)	103 (20)	108 (21)#^2^
SO_2_S [%]	GP	100 (0)	27 (25)*#^1^	80 (24)	96 (24)	93 (25)
	BEL	100 (0)	63 (17)*#^1^	98 (17)	105 (21)	107 (28)
	Control	100 (0)	65 (37)	82 (29)	112 (3)	151 (44)
SO_2_D [%]	GP	100 (0)	102 (17)	93 (21)	108 (19)	107 (26)
	BEL	100 (0)	96 (3)	101 (3)	101 (4)	99 (5)
	Control	100 (0)	106 (8)	106 (8)	106 (10)	113 (11)
FlowS [%]	GP	100 (0)	29 (28)*	109 (52)	116 (53)	110 (50)
	BEL	100 (0)	50 (25)*	139 (74)	157 (91)	183 (131)
	Control	100 (0)	84 (58)	120 (73)	155 (95)	189 (104)
FlowD [%]	GP	100 (0)	52 (50)*	189 (119)	201 (126)	190 (114)
	BEL	100 (0)	56 (22)*	121 (46)	126 (48)	131 (45)
	Control	100 (0)	68 (5)*	90 (4)	106 (19)	120 (16)
HbS [%]	GP	100 (0)	70 (15)*	78 (13)*	77 (14)*	79 (17)*
	BEL	100 (0)	85 (7)*	82 (16)*	89 (23)*	90 (27)*
	Control	100 (0)	76 (26)*	79 (26)*	89 (28)*	81 (15)*
HbD [%]	GP	100 (0)	84 (15)*	83 (12)	94 (15)	92 (14)
	BEL	100 (0)	98 (11)	90 (10)	91 (11)	88 (17)
	Control	100 (0)	105 (34)	174 (84)	146 (59)	152 (62)
VS [%]	GP	100 (0)	71 (22)*	99 (18)	101 (18)	103 (22)
	BEL	100 (0)	74 (16)*	114 (27)	118 (29)	120 (40)
	Control	100 (0)	84 (20)	85 (22)	84 (25)	77 (20)
VD [%]	GP	100 (0)	111 (122)	120 (17)	125 (15)	128 (20)
	BEL	100 (0)	74 (16)	114 (27)	118 (29)	120 (40)
	Control	100 (0)	105 (34)	174 (84)	146 (59)	152 (62)

*crSO*_*2*_ cerebral regional oxygen saturation, *SO*_*2*_*S* superficial oxygen saturation, *SO*_*2*_*D* deep oxygen saturation, *FlowS* superficial bloodflow, *FlowD* deep bloodflow, *HbS* superficial haemoglobin content, *HbD* deep haemoglobin content, VS superficial velocity, *VD* deep velocity

*indicates *p* < 0.05 vs. Baseline value. # indicates *p* < 0.05 in intergroup comparison (1 = colloid vs. crystalloid; 2 = colloid vs sham; 3 = crystalloid vs sham)

### Monitoring of cerebral oxygen saturation and peripheral microcirculation

We measured crSO_2_ with self-adherent near infrared spectroscopy sensors (SAFB-SM, Adult Soma Sensor, Covidien, USA) in real time, which were attached at the left and right forehead. These values were updated in five-second intervals by an INVOS™ 5100C Cerebral/Somatic Oximeter (Somanetics Corporation, USA), which calculates contributions from arterial and venous blood in a 1:3 ratio. This is a clinically established device for evaluation of crSO_2_ and used for extended cerebral monitoring particularly during cardiac and major vascular surgery [24–26]. Furthermore, the device may have a prognostic value in patients suffering from haemorrhagic shock [27, 28].

The O_2_C-device (LEA Medizintechnik GmbH, Germany) was used to evaluate local tissue oxygenation and microcirculatory perfusion by combining two separate approaches: laser-Doppler technique and the white-light-spectroscopy. Near infrared laser light and visible white light are emitted and detected. The laser-Doppler measures the Doppler-shift of the laser light (830 nm, 30 mW) that is caused by erythrocyte movement and allows for assessment of blood flow velocity. White-light-spectroscopy is used for measurement of oxygen saturation and the relative haemoglobin concentration in the investigated region. This method simultaneously detects up to 300 wavelengths of white light (450-850 nm, 30 mW).

Oxygenated and deoxygenated blood show different absorption ranges, which is used to calculate the oxygen saturation. The quantity of absorbed light is proportional to the haemoglobin content. Both light forms are emitted continuously and are scattered on the tissue surface. A fibre optic probe type LF-1 with a spatial resolution of 2 and 8 mm depth collects the light in a temporal resolution of 500 ms. This technique is used in s different areas of clinical application [29–33]. The probe was positioned into the animal’s snout to assess the peripheral microcirculation: buccal capillary oxygen saturation of haemoglobin, relative haemoglobin amount, blood flow and blood flow velocity.

### Experimental design

**Fig. 1:**
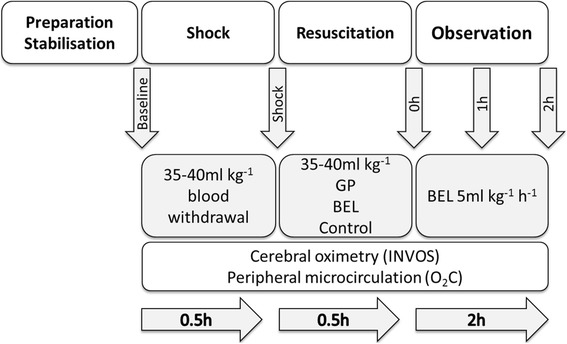
Experimental flow chart. GP: gelatin-polysuccinat; BEL: balanced electrolyte solution

The experimental protocol is displayed in fig. 1. After instrumentation and 30 min of consolidation, baseline measurements were documented and haemorrhagic shock was induced by means of arterial blood withdrawal over 15 min (35–40 ml kg^− 1^). A combined occurrence of decreased cardiac index (< 50% of the baseline value) and mean arterial pressure < 45 mmHg were required as shock criteria. After 30 min of shock, fluid resuscitation was started with rapid infusion of the individually removed amount of blood in a ratio of 1:1 by either GP (Gelafundin iso 4%, B. Braun, Germany, *n* = 9) or BEL (Sterofundin iso, B. Braun, Germany, n = 9) within 15 min. Nine animals served as control (untreated shock, n = 9). This approach was not influenced by specific cardio-circulatory parameter or goals. Per protocol both infusions differ in their components and biochemical properties (BEL: Na^+^ = 145.0 mmol/l, K^+^ = 4.0 mmol/l, Mg^2+^ = 1.0 mmol/l, Ca^2+^ = 2.5 mmol/l, Cl^−^ = 127.0 mmol/l, Acetat^−^ = 24.0 mmol/l, Malat^2−^ = 5.0 mmol/l, pH = 5.1–5.9, osmolarity = 309 mosm/l, GP gelatin-polysuccinat 40 g/1000 ml, Na^+^ = 154 mmol/l, Cl^−^ = 120 mmol/l, pH = 7.1–7.7, osmolarity = 274 mosm/l). Following the procedure, the animals were monitored for two hours. Subsequent to fluid resuscitation, BEL was infused at a rate of 5 ml kg^− 1^ h^− 1^. The animals did not receive any further fluid or catecholamine therapy. At the end of the observation, the animals were killed during deep general anaesthesia by central venous administration of propofol and potassium chloride.

### Statistics

The values are displayed as mean and standard deviation (SD) or plots. Key haemodynamic and microcirculation data are reported as percentages of the individual baseline. To analyse the effect between the three groups over time we performed a two-way analysis of variance (ANOVA) with pairwise multiple comparison correction using the Student-Newman-Keuls method. A *p* value lower than 0.05 was regarded as significant. The statistical evaluation was performed with the software package SigmaPlot 12.5 (Systat Software, USA).

## Results

**Fig. 2:**
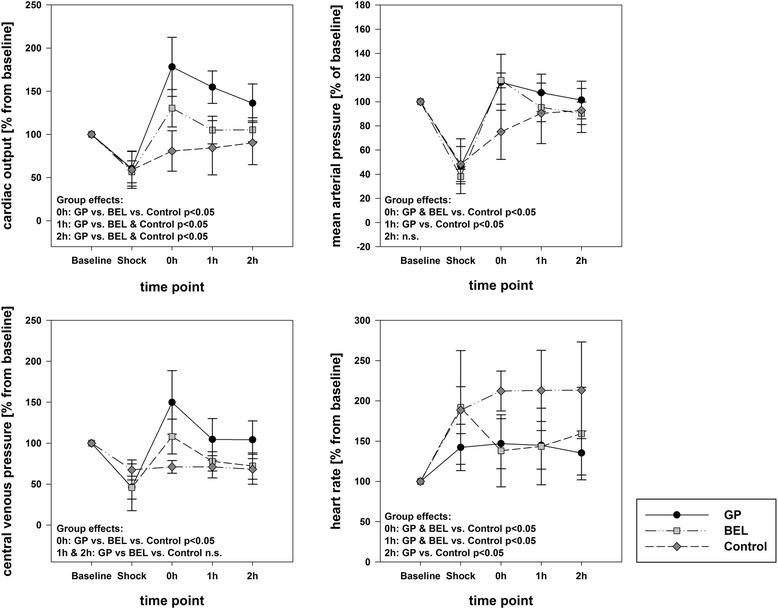
Key haemodynamic parameters. Group effects over time are analysed by two-way ANOVA and post-hoc Student-Newman-Keuls method; n.s. non-significant; GP: gelatin-polysuccinat; BEL: balanced electrolyte solution

**Fig. 3:**
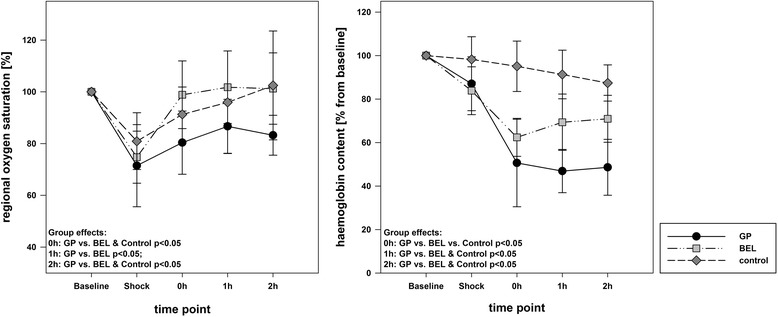
Cerebral oxygen saturation and haemoglobin content. Group effects over time are analysed by two-way ANOVA and post-hoc Student-Newman-Keuls method. GP: gelatin-polysuccinat; BEL: balanced electrolyte solution

**Fig. 4:**
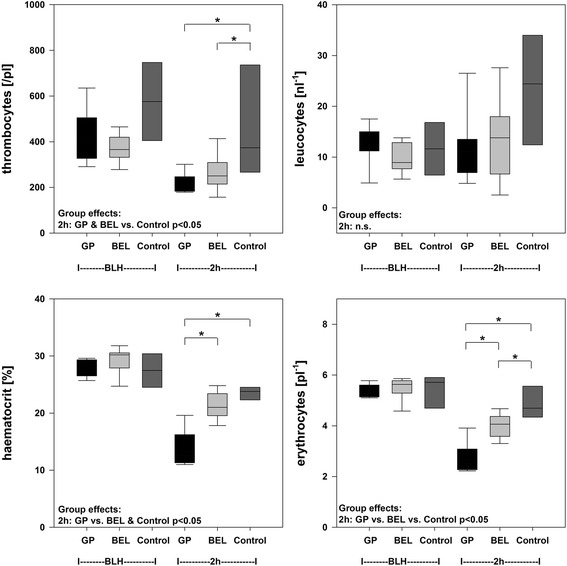
Haematological parameters. Group effects over time are analysed by two- way-ANOVA and post-hoc Student Newman-Keuls method. * = *p* < 0.05; n.s. non-significant; GP: gelatin-polysuccinat; BEL: balanced electrolyte solution

Haemorrhagic shock was induced by blood withdrawal of 1046 ± 61 ml (36.6 ± 2.1 ml kg^− 1^), which was comparable in all three groups. We observed a mean shock-like decrease of blood pressure (43 ± 16%), cardiac output (59 ± 16%) and haemoglobin content (87 ± 12%) in all animals. For fluid resuscitation we administered comparable amounts of GP (36.6 ± 1.7 ml kg^− 1^) and BEL (36.4 ± 1.7 ml kg^− 1^). Survival rates were 100% in both fluid groups, but only 33% in the control group. The key haemodynamic dataset is summarized in fig. 2 and documents comparable baseline and shock conditions in all three groups. Table 1 shows extended haemodynamic and blood gas parameters. Fluid resuscitation by means of both BEL and GP reliably stabilized the macrocirculation and re-achieved, or surpassed the baseline conditions: infusion of GP initially led to significantly higher cardiac output (178 ± 32% (GP) vs. 130 ± 20% (BEL) vs. 81 ± 19% (control); each *p* < 0.01), global enddiastolic volume, central venous pressure, and central venous oxygen saturation. This was not reflected in higher mean arterial pressure or lower heart rate. Within two hours, the GP and BEL groups re-assimilate in terms of macrocirculation. Additionally, the three surviving control animals tended to stabilize without treatment, which was associated with persisting tachycardia. Haemorrhagic shock conditions significantly impaired the crSO_2_. In the control and BEL groups, baseline conditions were re-achieved within two hours. In the GP group, impaired crSO_2_ persisted significantly differing from the other groups (fig. 3). Peripheral tissue oxygenation and blood flow data adequately reproduced the early shock-induced microcirculatory impairment, but do not differed between the groups over time (Table 2). Following fluid resuscitation, the blood cell counts considerably drop (figs. 3, 4). Haemoglobin, haematocrit and thrombocyte counts were significantly lower in the GP group.

## Discussion

This study compares GP and BEL for early treatment of haemorrhagic shock in a porcine model with focus on cerebral oxygenation and peripheral microcirculation. Fluid resuscitation by comparable amounts of GP and BEL were equipotent in re-achieving the pre-shock state of macro- as well as microcirculatory parameters. GP administration caused a persisting impairment of the crSO_2_. We observed persisting crSO_2_ deterioration with an approximate 20% decrease from the individual baseline value. In clinical scenarios, drops of 20–25% describe a red line that may require urgent intervention to minimize cerebral ischaemia [34–36]. In perioperative settings, these kinds of deoxygenation events are associated with neurocognitive dysfunction, cerebral ischaemia and morbidity [34–36].

Early fluid resuscitation to treat a haemorrhagic shock primarily needs to focus on rapid stabilisation of the systemic circulation, and in particular the mean arterial pressure to warrant perfusion and oxygen supply of vital organs. However, macrocirculation alone represents only a poor surrogate for microcirculatory and organ perfusion [37]. We simulated an immediate but not ongoing haemorrhage, i.e. an acute blood loss during surgery with consecutive bleeding control: a composite of high volume blood loss (about 50% of the circulating blood volume) and a shock-like haemodynamic deterioration was used to define shock criteria, which comes below the acceptable thresholds in current permissive hypotension concepts [6]. Both fluid regimes stabilized the haemodynamics and at least re-achieved baseline state. These effects were more pronounced following GP administration with significant lower heart rate, higher cardiac output, global enddiastolic volume, and central venous pressure, which can be explained by the specific properties of colloids leading to higher intravascular volume and colloid-osmotic pressure [12]. Several experimental studies assessed the effects of various colloids and especially GP on distinct end organ sites. Comparable time profiles were also found in postoperative hypovolaemic patients receiving fluid resuscitation [38]. Maier et al. compared different colloids in a porcine haemorrhagic shock model over thirty minutes, and further re-transfused the extracted blood [39]. They report an increase of peripheral tissue oxygen saturation and microcirculatory re-compensation following HES as well as GP infusion. This is consistent with our data, though also realized by BEL administration and the surviving untreated animals, possibly through endogenous compensation mechanisms. The impact on the brain as a highly vulnerable organ has been rarely examined. The cerebral microcirculation shows a different behaviour than the peripheral one and is preserved to a certain degree during haemodynamic deterioration through blood loss by blood flow redistribution [40]. In pigs, cerebral near infrared spectroscopy adequately depicts haemorrhage-induced, cardio-circulatory collapse with a time delay of five to nine minutes [41] that possibly reflects this compensatory blood flow redistribution to maintain cerebral perfusion. Non-invasive crSO_2_ measurement can be affected by extracerebral tissue. However, this technology is widely accepted in clinical and experimental settings. Particularly in pigs, significant correlations between crSO_2,_ brain tissue partial oxygen pressure, quantitative electroencephalography and cerebral venous oxygen saturation were shown [42, 43].

The recent uncertainty regarding the administration of HES has led to increased use of gelatin-based colloids in the last years. Beyond relevant rates of anaphylaxis, the risk profile of GP remains inconclusive due to lack of high quality data [16]. Another meta-analysis found a favourable renal risk profile in comparison to older HES solutions [44]. Theoretically, crystalloid to colloid ratios of about 1:1.5 may achieve similar haemodynamic effects, but exhibit considerable individual heterogeneity independently of the type of colloid [8]. The present model in this context chose a complete and 1:1 replacement of the drawn blood leading to macrocirculatory stabilization beyond physiological baseline characteristics in the GP group. This approach, however, appears realistic in the clinical routine because ongoing fluid therapy is hardly stopped immediately upon bleeding control and after reversal of severe haemorrhagic shock. Furthermore, sufficiency of bleeding control and availability of allogeneic blood products is limited in the early phase. The consequence was considerable haemodilution through GP and is documented in the significantly lowered haemoglobin and haematocrit counts. Moreover, we found signs of impaired cerebral oxygen delivery. Beyond indicating impending haemodynamic decompensation [41], the early use of non-invasive cerebral near-infrared spectroscopy may therefore help to detect inadequate organ oxygen supply despite re-established macrocirculation and even indicate over-treatment. BEL administration and even the surviving untreated control animals displayed higher crSO_2_ values. Hence, also BEL can be efficient for reversal of severe haemorrhagic shock, if bleeding is controlled. Non-treatment and permissive acceptance of cardio-circulatory failure is not a reliable option, and well document in the low survival rate: six of nine untreated animals died before finishing the protocol, and were not replaced for ethical as well as experimental reasons. Forced or uncritical fluid resuscitation by GP appears to be a double-edged sword, because the pronounced volume and haemodynamic effect may even cause further harm due to unrecognized crSO_2_ impairment. Furthermore, current European Guidelines restrict the use of colloids in major bleeding scenarios due to deterioration of haemostasis [6].

The present study has some limitations: due to the lack of adequate data for a sample size calculation the present studies served as pilot data, whereas we adapted the sample size from previous large animal studies focussing on acute cardiorespiratory effects [45]. The short-term protocol and pilot character of the study did not allow for extended neurological testing or assessment of brain damage. Further studies will have to focus on neuroinflammatory and cognitive aspects of fluid resuscitation and different solutions. Therefore, we can only speculate on potential risk for hypoxaemic brain damage in the long run. The correlation between crSO_2_ impairment and adverse patient outcome, however, is well examined [34–36]. For experimental reasons, we choose a scenario without any vasopressor support to address the mere effects of fluid resuscitation. Furthermore, no blood re-transfusion was conducted to focus particularly on the early phase of haemorrhagic shock before availability of extended interventions like transfusion of blood products or specific and goal-directed treatment of coagulation disorders. The use of pigs allows for a setting that is comparable with clinical practise and the physiological response is very similar to humans. The mean porcine haemoglobin content (8.9 ± 1.1 g/dl in this study), however significantly differs from human values [46]. The three experimental groups significantly differed in the applied respiratory minute volume through adaption of the respiratory rates, whereby the three surviving untreated animals were slightly hyperventilated (Table 1). However, given the fully comparable and non-differing parameters that indicate sufficient gas exchange (PaO_2_, PaCO_2_, PaO_2_/FiO_2_) the relevancy of this finding is low.

## Conclusion

Fluid resuscitation of severe haemorrhagic shock with GP was not superior to an equal amount of BEL. Despite more pronounced effects on systemic circulation, GP neither ameliorated microcirculatory perfusion nor was more favourable in restoring crSO_2_ compared to BEL. Forced fluid resuscitation by GP should be applied with caution to prevent haemodilution-induced impairment of cerebral oxygen delivery. Early crSO_2_ measurement may prevent unrecognized episodes of impairment through forced fluid resuscitation.

## Abbreviations

BEL: balanced electrolyte solution
crSO_2_: cerebral regional oxygen saturation
GP: gelatin-polysuccinat
HES: hydroxyethyl starch
PiCCO: pulse contour cardiac output system
SD: standard deviation

## References

[1] Kutcher ME, Kornblith LZ, Narayan R, Curd V, Daley AT, Redick BJ (2013). A paradigm shift in trauma resuscitation: evaluation of evolving massive transfusion practices. JAMA Surg.

[2] Noll E, Diana M, Charles AL, Singh F, Gan TJ, Pottecher J (2017). Comparative analysis of resuscitation using human serum albumin and crystalloids or 130/0.4 hydroxyethyl starch and crystalloids on skeletal muscle metabolic profile during experimental haemorrhagic shock in swine: a randomised experimental study. Eur J Anaesthesiol.

[3] Nielsen TK, Hvas CL, Dobson GP, Tonnesen E, Granfeldt A (2014). Pulmonary function after hemorrhagic shock and resuscitation in a porcine model. Acta Anaesthesiol Scand.

[4] Bogert JN, Harvin JA, Cotton BA (2016). Damage Control Resuscitation. J Intensive Care Med.

[5] Tanczos K, Nemeth M, Molnar Z (2015). What's new in hemorrhagic shock?. Intensive Care Med.

[6] Rossaint R, Bouillon B, Cerny V, Coats TJ, Duranteau J, Fernandez-Mondejar E, et al. the European guideline on management of major bleeding and coagulopathy following trauma: fourth edition. Critical care (London, England) 2016;20:100. 10.1186/s13054-016-1265-x PMC4828865 27072503

[7] Kozek-Langenecker SA, Afshari A, Albaladejo P, Santullano CA, De Robertis E, Filipescu DC (2013). Management of severe perioperative bleeding: guidelines from the European Society of Anaesthesiology. Eur J Anaesthesiol.

[8] Orbegozo Cortes D, Gamarano Barros T, Njimi H, Vincent JL (2015). Crystalloids versus colloids: exploring differences in fluid requirements by systematic review and meta-regression. Anesth Analg.

[9] Guidet B, Martinet O, Boulain T, Philippart F, Poussel JF, Maizel J (2012). Assessment of hemodynamic efficacy and safety of 6% hydroxyethylstarch 130/0.4 vs. 0.9% NaCl fluid replacement in patients with severe sepsis: the CRYSTMAS study. Critical care (London, England).

[10] Brunkhorst FM, Engel C, Bloos F, Meier-Hellmann A, Ragaller M, Weiler N (2008). Intensive insulin therapy and pentastarch resuscitation in severe sepsis. N Engl J Med.

[11] Perner A, Haase N, Guttormsen AB, Tenhunen J, Klemenzson G, Aneman A (2012). Hydroxyethyl starch 130/0.42 versus Ringer's acetate in severe sepsis. N Engl J Med.

[12] Annane D, Siami S, Jaber S, Martin C, Elatrous S, Declere AD (2013). Effects of fluid resuscitation with colloids vs crystalloids on mortality in critically ill patients presenting with hypovolemic shock: the CRISTAL randomized trial. JAMA.

[13] Dellinger RP, Levy MM, Rhodes A, Annane D, Gerlach H, Opal SM (2013). Surviving sepsis campaign: international guidelines for management of severe sepsis and septic shock, 2012. Intensive Care Med.

[14] Myburgh JA, Finfer S, Bellomo R, Billot L, Cass A, Gattas D (2012). Hydroxyethyl starch or saline for fluid resuscitation in intensive care. N Engl J Med.

[15] Feldheiser A, Pavlova V, Bonomo T, Jones A, Fotopoulou C, Sehouli J (2013). Balanced crystalloid compared with balanced colloid solution using a goal-directed haemodynamic algorithm. Br J Anaesth.

[16] Moeller C, Fleischmann C, Thomas-Rueddel D, Vlasakov V, Rochwerg B, Theurer P (2016). How safe is gelatin? A systematic review and meta-analysis of gelatin-containing plasma expanders vs crystalloids and albumin. J Crit Care.

[17] Ertmer C, Rehberg S, Van Aken H, Westphal M (2009). Relevance of non-albumin colloids in intensive care medicine. Best Pract Res Clin Anaesthesiol.

[18] Beyer R, Harmening U, Rittmeyer O, Zielmann S, Mielck F, Kazmaier S (1997). Use of modified fluid gelatin and hydroxyethyl starch for colloidal volume replacement in major orthopaedic surgery. Br J Anaesth.

[19] Awad S, Dharmavaram S, Wearn CS, Dube MG, Lobo DN (2012). Effects of an intraoperative infusion of 4% succinylated gelatine (Gelofusine(R)) and 6% hydroxyethyl starch (Voluven(R)) on blood volume. Br J Anaesth.

[20] Qureshi SH, Rizvi SI, Patel NN, Murphy GJ (2016). Meta-analysis of colloids versus crystalloids in critically ill, trauma and surgical patients. Br J Surg.

[21] National Clinical Guideline C. National Institute for Health and Clinical Excellence: Guidance. Intravenous Fluid Therapy: Intravenous Fluid Therapy in Adults in Hospital. London: Royal College of Physicians (UK) National Clinical Guideline Centre.; 2013.

[22] Wu CY, Chan KC, Cheng YJ, Yeh YC, Chien CT (2015). Effects of different types of fluid resuscitation for hemorrhagic shock on splanchnic organ microcirculation and renal reactive oxygen species formation. Critical care (London, England).

[23] Witt L, Glage S, Lichtinghagen R, Pape L, Boethig D, Dennhardt N (2016). Impact of high doses of 6% hydroxyethyl starch 130/0.42 and 4% gelatin on renal function in a pediatric animal model. Paediatr Anaesth.

[24] Edmonds HL, Ganzel BL, Austin EH (2004). Cerebral oximetry for cardiac and vascular surgery. Semin Cardiothorac Vasc Anesth.

[25] Murkin JM, Adams SJ, Novick RJ, Quantz M, Bainbridge D, Iglesias I (2007). Monitoring brain oxygen saturation during coronary bypass surgery: a randomized, prospective study. Anesth Analg.

[26] Hong SW, Shim JK, Choi YS, Kim DH, Chang BC, Kwak YL (2008). Prediction of cognitive dysfunction and patients' outcome following valvular heart surgery and the role of cerebral oximetry. Eur J Cardiothorac Surg.

[27] Al Tayar A, Abouelela A, Mohiuddeen K. Can the cerebral regional oxygen saturation be a perfusion parameter in shock? J Crit Care 2017;38:164–167. 10.1016/j.jcrc.2016.11.006 27915164

[28] Torella F, Cowley RD, Thorniley MS, McCollum CN (2002). Regional tissue oxygenation during hemorrhage: can near infrared spectroscopy be used to monitor blood loss?. Shock (Augusta, Ga).

[29] Klein KU, Schramm P, Glaser M, Reisch R, Tresch A, Werner C (2010). Intraoperative monitoring of cerebral microcirculation and oxygenation--a feasibility study using a novel photo-spectrometric laser-Doppler flowmetry. J Neurosurg Anesthesiol.

[30] Knobloch K, Lichtenberg A, Pichlmaier M, Mertsching H, Krug A, Klima U (2003). Microcirculation of the sternum following harvesting of the left internal mammary artery. Thorac Cardiovasc Surg.

[31] Ubbink R, Bettink MAW, Janse R, Harms FA, Johannes T, Munker FM (2017). A monitor for cellular oxygen METabolism (COMET): monitoring tissue oxygenation at the mitochondrial level. J Clin Monit Comput.

[32] Abel G, Allen J, Drinnan M (2014). A pilot study of a new spectrophotometry device to measure tissue oxygen saturation. Physiol Meas.

[33] Martini M, Rohrig A, Wenghoefer M, Schindler E, Messing-Junger AM (2014). Cerebral Oxygenation and hemodynamic measurements during craniosynostosis surgery with near-infrared spectroscopy. Childs Nerv Syst.

[34] Yao FS, Tseng CC, Ho CY, Levin SK, Illner P (2004). Cerebral oxygen desaturation is associated with early postoperative neuropsychological dysfunction in patients undergoing cardiac surgery. J Cardiothorac Vasc Anesth.

[35] Slater JP, Guarino T, Stack J, Vinod K, Bustami RT, Brown JM (2009). Cerebral oxygen desaturation predicts cognitive decline and longer hospital stay after cardiac surgery. Ann Thorac Surg.

[36] Brodt J, Vladinov G, Castillo-Pedraza C, Cooper L, Maratea E (2016). Changes in cerebral oxygen saturation during transcatheter aortic valve replacement. J Clin Monit Comput.

[37] Gruartmoner G, Mesquida J, Ince C (2015). Fluid therapy and the hypovolemic microcirculation. Curr Opin Crit Care.

[38] Gondos T, Marjanek Z, Ulakcsai Z, Szabo Z, Bogar L, Karolyi M (2010). Short-term effectiveness of different volume replacement therapies in postoperative hypovolaemic patients. Eur J Anaesthesiol.

[39] Maier S, Holz-Holzl C, Pajk W, Ulmer H, Hengl C, Dunser M (2009). Microcirculatory parameters after isotonic and hypertonic colloidal fluid resuscitation in acute hemorrhagic shock. J Trauma.

[40] Wan Z, Sun S, Ristagno G, Weil MH, Tang W (2010). The cerebral microcirculation is protected during experimental hemorrhagic shock. Crit Care Med.

[41] Navarro LH, Lima RM, Khan M, Dominguez WG, Voigt RB, Kinsky MP (2012). Continuous measurement of cerebral oxygen saturation (rSO(2)) for assessment of cardiovascular status during hemorrhagic shock in a swine model. The journal of trauma and acute care surgery.

[42] Putzer G, Braun P, Strapazzon G, Toferer M, Mulino M, Glodny B (2016). Monitoring of brain oxygenation during hypothermic CPR - a prospective porcine study. Resuscitation.

[43] Weenink RP, Hollmann MW, Stevens MF, Kager J, van Gulik TM, van Hulst RA (2014). Detection of cerebral arterial gas embolism using regional cerebral oxygen saturation, quantitative electroencephalography, and brain oxygen tension in the swine. J Neurosci Methods.

[44] Saw MM, Chandler B, Ho KM (2012). Benefits and Risks of using gelatin solution as a plasma expander for perioperative and critically ill patients: a meta-analysis. Anaesth Intensive Care.

[45] Ziebart A, Hartmann EK, Thomas R, Liu T, Duenges B, Schad A, et al. Low tidal volume pressure support versus controlled ventilation in early experimental sepsis in pigs. Respir Res. 2014;15(101) 10.1186/s12931-014-0101-6 PMC4172867 25189285

[46] Hahn RG (2013). Fluid therapy in uncontrolled hemorrhage--what experimental models have taught us. Acta Anaesthesiol Scand.

